# Human Papillomaviruses as Infectious Agents in Gynecological Cancers. Oncogenic Properties of Viral Proteins

**DOI:** 10.3390/ijms23031818

**Published:** 2022-02-05

**Authors:** Daria A. Haręża, Jacek R. Wilczyński, Edyta Paradowska

**Affiliations:** 1 Laboratory of Virology, Institute of Medical Biology of the Polish Academy of Sciences, 93-232 Lodz, Poland; dhareza@cbm.pan.pl; 2 BioMedChem Doctoral School of the University of Lodz and Lodz Institutes of the Polish Academy of Sciences, 90-237 Lodz, Poland; 3 Department of Surgical and Oncological Gynecology, Medical University of Lodz, 90-419 Lodz, Poland; jrwil@post.pl

**Keywords:** gynecological cancers, human papillomavirus, oncoprotein

## Abstract

Human papillomaviruses (HPVs), which belong to the *Papillomaviridae* family, constitute a group of small nonenveloped double-stranded DNA viruses. HPV has a small genome that only encodes a few proteins, and it is also responsible for 5% of all human cancers, including cervical, vaginal, vulvar, penile, anal, and oropharyngeal cancers. HPV types may be classified as high- and low-risk genotypes (HR-HPVs and LR-HPVs, respectively) according to their oncogenic potential. HR-HPV 16 and 18 are the most common types worldwide and are the primary types that are responsible for most HPV-related cancers. The activity of the viral E6 and E7 oncoproteins, which interfere with critical cell cycle points such as suppressive tumor protein p53 (p53) and retinoblastoma protein (pRB), is the major contributor to HPV-induced neoplastic initiation and progression of carcinogenesis. In addition, the E5 protein might also play a significant role in tumorigenesis. The role of HPV in the pathogenesis of gynecological cancers is still not fully understood, which indicates a wide spectrum of potential research areas. This review focuses on HPV biology, the distribution of HPVs in gynecological cancers, the properties of viral oncoproteins, and the molecular mechanisms of carcinogenesis.

## 1. Introduction

Viral infections are recognized as strong risk factors for some types of cancer. Human papillomavirus (HPV) is the most common sexually transmitted infection and occurs via direct skin-to-skin or mucosa-to-mucosa contact. Benign cutaneous manifestations of HPV usually include papillomas, which are commonly called warts. HPV can act as a direct carcinogen by infecting cells that subsequently undergo neoplastic transformation. It exhibits specific tropism for the squamous epithelium. Specific types of the virus grow in the skin, while others grow in mucous membranes such as the vagina. The clinical effect of infection is skin and mucosal lesions in the form of warts and condylomas and in the form of both low-grade and high-grade dysplasia, the latter being a premalignant lesion. Worldwide, approximately 690,000 of the new cancer cases that are diagnosed every year can be attributed to HPV infection (age-standardized incidence rate (ASIR) of 8.0 cases per 100,000 person-years) [1]. Moreover, HPV is the second leading infectious cause of cancer following *Helicobacter pylori*. Among the 690,000 HPV-attributable cancer cases, 570,000 (83%) are cervical cancer cases, of which 500,000 (72%) can be attributed to the high-risk HPV (HR-HPV) types 16 and 18 and 120,000 (17%)—to HPV types 31, 33, 45, 52, and 58. HPV16 and HPV18 are responsible for almost all HPV-related cancers in men [1]. Epidemiological studies show the detection of HPV in almost all cases of cervical cancer and in the majority of vaginal and vulvar carcinomas [2]. Walboomers et al. showed that the HPV genome is found in 99.7% of cervical squamous cell carcinoma samples [3]. Another study comprising 14,249 cases of invasive cervical cancer collected from 38 countries worldwide revealed that HR-HPV types 16 and 18 are detected in 71% of invasive cervical cancers worldwide, while HPV 16, 18, and 45 are the predominant types in cervical adenocarcinomas [4]. The other frequently identified virus types in invasive cervical cancers are HPV 31, 33, 35, 45, 52, and 58 which demonstrate slight prevalence variations depending on the geographic distribution [4]. HPV DNA and proteins are also detected in cancerous ovarian tissues and fallopian tube samples [5,6]. However, the role of HPV in ovarian and endometrial malignancies is still controversial.

The review provides new insight into the pathogenesis of HPV-related gynecological cancers and the possible HPV participation in the development of ovarian cancer. This review also discusses morphological and genetic aspects of HPV biology, oncogenic properties of viral proteins, and their effect on signaling pathways. To fully understand the HPV role in gynecological neoplasms, it seems appropriate to begin our review by discussing the biology of HPV and the function of its oncoproteins.

## 2. HPV Genome and Proteins

### 2.1. Genome

**Figure 1:**
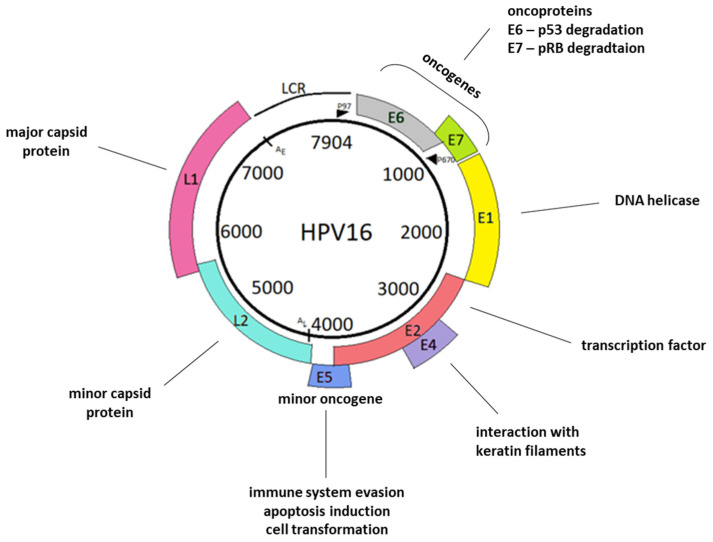
Schematic representation of the HPV16 genome. The viral genome consists of the *L1* and *L2* genes, encoding major capsid protein L1, minor capsid protein L2, and the long regulatory region (LCR). LCR is the least conserved genome region. It contains a p97 promoter and numerous sequences that function as enhancers and silencers of viral transcription. The remaining HPV genome sequences comprise early genes: *E1*, *E2*, *E4*, *E5*, *E6*, and *E7*. E1 participates in viral DNA replication. E2 functions in transcriptional control, tethering of viral episomes, and, similarly to E1, participates in viral DNA replication. E4 interact with the cytoskeleton, while E5 participates in genome amplification. E6 and E7 interact with tumor suppressor proteins. Furthermore, both E5 and E6, as well as E7, also have many different functions, which are described in the “Oncoproteins” section. In the viral genome, we also distinguish late polyadenylation sites, AL; early polyadenylation sites, AE; and a late promoter, P670 [15].

Human papillomaviruses are small nonenveloped viruses that belong to the *Papillomaviridae* family. The HPV genome is a circular double-stranded DNA molecule approximately 8000 bp in length and associated with histones to create chromatin-like structures [7]. HPVs have an icosahedral virion consisting of two structural proteins: 360 copies of the 55 kDa L1 protein and 12 copies of the 74 kDa L2 protein [8,9]. The genome encodes eight open reading frames (ORFs) that are arranged on one DNA strand. The genome is polycistronic, and several types of alternative splicing mechanisms generate viral mRNAs [10]. In the HPV genome, three regions are distinctive: early (E)—encodes nonstructural proteins of the virus; late (L)—encodes structural proteins; and a long regulatory or noncoding region (LCR or NCR, respectively) (Figure 1). The E region contains more than 50% of the genome, while the L region represents 40% of the genome [11]. The E and L regions are separated by two polyadenylation sites: early AE and late AL. The most conserved fragment in the HPV genome is the region coding the E1 and L1 proteins. This conservation is the reason why HPV taxonomy is based on the nucleotide sequence of the ORF coding for the capsid protein L1 [12]. HPV types and genotypes are distinguished based on at least a 10% difference within the *L1* gene sequence. More than 200 different HPV genotypes have been identified that are categorized according to their epidemiologic association with cancer. Isolates of a virus type, whose *L1* genes differ from an established type by 2–10%, as well as intermediates between types and variants are considered subtypes [13]. Genomes varying from the reference strain DNA sequence by ~2% or less are termed variants of the closest HPV type [14,15]. In the taxonomy context, LCR sequences, constituting the least conserved fragment of the HPV genome, have been used most often to describe intertype diversity, i.e. the relationship between variants, but recent studies have shown that the early–late intergenic region and the *E4* and *E5* genes are also hypervariable [16]. With respect to the genome structure, approximately twenty protein factors that bind to specific sequence elements in the LCR have been identified, including activating protein-1 (AP-1) [17], octamer binding factor-1 (OCT-1), papillomavirus enhancer factor-1 (PEF-1), transcription enhancer factor-1 and -2 (TEF-1 and TEF-2, respectively) [18]. The next very important part of the genome is the region that encodes the early genes of the virus. These genes encode only six regulatory proteins that are critical for the maintenance of the viral genome in the cell, its replication, and the activation of the lytic cycle [19]. 

HPV genotypes are divided into two groups: oncogenic HR-HPV and non-cancerous low-risk (LR) types. The oncogenic group includes 14 HR-HPV types: 16, 18, 31, 33, 35, 39, 45, 51, 52, 56, 58, 59, 66, 68; and 23 LR-HPV types: HPV 6, 11, 26, 40, 42, 53, 54, 55, 61, 62, 64, 67, 69, 70, 71, 72, 73, 81, 82, 83, 84, IS39, and CP6108 [20]. HPV16 is the most prevalent HR-HPV worldwide and causes the majority of cancer cases [21,22]. Both the HPV16 and HPV18 types are mainly associated with the malignant progression of cervical tumors and other cancers of genital organs [23].

### 2.2. Early and Late Viral Proteins

Early proteins E1 and E2 are encoded by the E1 and E2 ORFs and are known to regulate the replication of the viral genome, as well as transcription of early proteins. These viral proteins tightly control and maintain the expression of the E6 and E7 oncoproteins at low levels. In particular, the E2 protein represses transcription of the *E6* and *E7* oncogenes through its specific binding to DNA recognition sites located within the promoter sequences. Loss of the E2 repressive functions may result in deregulated expression of both oncogenes and initiation of the transformation process [24]. The E1 and E2 proteins are also critical for episomal copy number maintenance of the viral genome. Viral integration leads to the linearization of the HPV genome, usually in the region of the *E1* and *E2* genes, and the possibility of partial or total deletion of these genes [25]. The loss of these genes leads to the overexpression of the *E6* and *E7* genes and contributes to oncogenesis. The E4 ORF lies within the E2 ORF, but *E4* gene products are expressed from the E1∧E4 spliced mRNA [26]. The E4 protein function is cell cycle arrest and disruption of keratin filaments [27]. It is also suggested that it may facilitate efficient viral release and transmission. Because the E4 protein is deposited as amyloid fibers, it can be used as an infection biomarker of active virus infection and disease severity [26,28]. Proteins E5, E6, and E7 which play important roles in the tumorigenesis process are described below in the “Oncoproteins” section.

The two late genes encode L1 (major) and L2 (minor) proteins, which form an icosahedral capsid around the HPV genome during the generation of progeny virions. The L1 protein has DNA-binding activity, while the L2 protein has domains capable of interacting with L1 capsomeres. During infectious entry, the nonenveloped virion uncoats in the endosome, whereupon conformational changes result in a dissociation of L1 from L2, which remains in complex with the HPV DNA. Capsid proteins L1 and L2 are critical for virion assembly [29]. The L1 proteins are firstly synthesized in the cytoplasm and after that are transported to the nucleus to package viral chromatin. The L2 protein binds specific sites of viral DNA in the nucleus and recruits L1 for new viral particles to be assembled [22]. It was assumed that the L2 protein also mediates the egress of the viral genome from endosomes [30]. Both capsid proteins are also involved in important interactions with cellular macromolecules that facilitate viral entry into keratinocytes. The more detailed properties and functions of all HPV proteins are presented in Table 1.

### 2.3. Oncoproteins

Three early HPV proteins, E6, E7, and E5, play an important role in the process of oncogenesis. The E6 and E7 proteins are related to functional inactivation of the main regulators of the cell cycle, tumor transformation suppressors, and activation of telomerases, while E5 enables keratinocyte differentiation and immune evasion [29,66]. HR-HPV oncoproteins cooperate to enhance malignant transformation [52].

#### 2.3.1. E6 Protein

**Figure 2:**
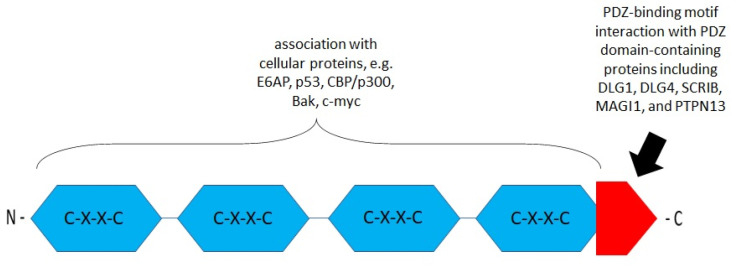
Schematic structure of the E6 oncoprotein (based on Boulet G. et al., 2007) [71]. The E6 protein contains four CXXC motifs (blue). The functions of these motifs are associated with cellular proteins, transcriptional activation, transformation, and immortalization. The E6 carboxy-terminal domain contains a PDZ (PSD95/DLG/ZO-1-)-binding motif (red) engaged in the interaction with PDZ domain-containing proteins such as discs, large homolog 1 (DLG1), DLG4, scribble planar cell polarity protein (SCRIB), membrane-associated guanylate kinase (MAGI1), and tyrosine–protein phosphatase non-receptor type 13 (PTPN13) [10,71,72,73].

The E6 oncoprotein in some types of cancer is involved in carcinogenesis. It may participate in transcriptional activation, transformation, and immortalization or associate with cellular proteins. The E6 protein acts as a repressor of apoptosis and promotes the survival of severely damaged cells. The E6 protein consists of approximately 150 amino acid residues coding an 18 kDa protein [46]. It contains two zinc finger-binding domains near four Cys–X–X–Cys (CXXC) motifs (Figure 2) [67]. The PDZ-binding motif in the carboxy-terminal domain is critical for its interactions with cellular proteins [68]. In vitro studies identified proteins with PDZ domains to which the E6 PDZ-binding motif can bind, including the scribble planar cell polarity protein (SCRIB) and discs, large homologs (DLGs) [69,70]. E6 binds to the cellular E3 ligase, E6-associating protein (E6AP), and this heterodimer then targets p53 for degradation via the ubiquitin–proteasome pathway. This process inhibits p53-dependent signaling and affects the control of cell cycle progression, contributing to tumorigenesis [47]. E6AP is a member of *hect* (homologous to the E6-associated protein carboxy-terminal domain) domain E3 ligases [52]. E6AP contributes to the increased stability of HPV16 and HPV18 E6 independently of E6AP’s catalytic activity [54].

#### 2.3.2. E7 Protein

**Figure 3:**
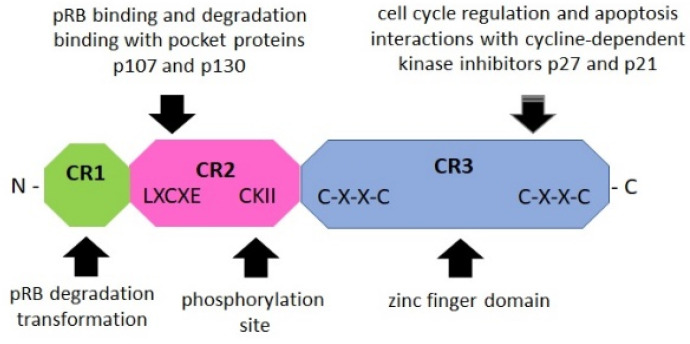
Schematic structure of the E7 oncoprotein (based on Boulet G. et al., 2007) [71]. The E7 oncoprotein contains three conserved regions (CR1/2/3). The NH2-terminal CR1 domain (green) is necessary for cellular transformation and pRB degradation but does not directly contribute to pRB binding. This domain interacts with host proteins such as E3 ubiquitin-protein ligase UBR4/p600 and p300/CBP-associated factor (PCAF), also known as K (lysine) acetyltransferase 2B [75]. The CR2 domain (pink) contains the pRB-binding core sequence LXCXE and a phosphorylation site for casein kinase II (CKII). The COOH-terminal CR3 domain (blue) is conserved and encodes a zinc finger domain containing two copies of the CXXC motif. This region is implicated in the association of pRB and other host cellular proteins. It is also critical for zinc-dependent dimerization and for mediating E7 interactions with cellular proteins crucial for cell cycle regulation and apoptosis (p21 and pRB) [10,71,75,76].

The E7 oncoprotein is a phosphoprotein of approximately 100 amino acids that contains three conserved regions 1/2/3 (CR1/2/3). The CR3 region at the carboxyl terminus encodes a zinc finger domain containing two CXXC motifs (Figure 3). The CR2 region contains a conserved LXCXE domain that binds to the “pocket domains” of pRB and suppresses its tumor suppressor activity [71,74]. The CR1 domain is necessary for pRB degradation and cellular transformation [71]. E7 functions as a promoter for replication and cell growth.

The E6 and E7 oncoproteins are essential components for cellular immortalization and transformation, as well as carcinogenesis induced by HPV. The interactions of the HPV oncoproteins with host cellular proteins are involved in the activation or repression of cell cycle progression in carcinogenesis. The oncoproteins’ common function gives rise to a complementary and synergistic effect, inducing an increase in transforming activity [77]. The integration of the viral genome into the host genome and high expression of the E6 and E7 proteins leads to neoplastic transformation and the development of some cancers. 

#### 2.3.3. E5 Protein

The *E5* gene encoding the E5 protein is expressed early during the lytic cycle of HPV. This gene is frequently deleted when the viral genome is integrated into the DNA of a host cell during malignant progression [78,79]. The E5 protein is an 83 amino acid hydrophobic protein associated with the Golgi, cytoplasmic, and endosomal membranes [74]. The molecular weight of E5 is 9.4 kDa for HPV16 and 8.3 kDa for HPV18 [80]. This protein folds in three putative hydrophobic regions with α-helical structure, which probably function as transmembrane domains. It interacts with integral membrane proteins to perform the functions of a proton pump. E5 plays an important role in cell signaling modulation through its association with a vacuole proton ATPase, decreasing the endosomal acidification that normally leads to the degradation of cell surface receptors. Dysregulation of endosomal acidification causes decreased turnover of cell surface receptors and increases their signaling activity [81]. What is more, E5 is probably the major transforming protein of bovine papillomavirus (BPV). E5 mediates BPV effects on cell behavior via an association with the PDGF receptor (PDGFR), resulting in constitutive activation of PDGF signaling [44]. It is important to point out that the HPV E5 protein shows little homology with BPV E5 and does not associate with the PDGFR. E5 proteins are weakly oncogenic and are most likely not directly involved in carcinogenesis, but further insights into the transforming abilities of E5 showed that E5 may enhance the oncogenic abilities of the major transforming proteins E6 and E7 in animal model studies [82,83]. 

Expression of the E5 protein disrupts the synthesis and function of the major histocompatibility complex (MHC) class I and II proteins. MHC class I downregulation can promote immune cell evasion by precluding cytotoxic T lymphocytes from recognizing infected cells [84]. E5 may participate in neoplastic transformation of cells by regulating the expression of other viral proteins; for example, E5 can be critical to cell transformation when it interacts with E6. In cultures of human cervical cells in vitro, E5, in conjunction with E6, is critical for the formation of koilocytes, which are morphological markers of HPV infection [85]. Most likely, koilocytes are created due to E5-induced translocation of calpactin I to the perinuclear region, which promotes perinuclear membrane fusion [45]. Barbaresi et al. [86] demonstrated the role of E5 in a human keratinocyte model (HaCaT cell line). These studies have shown that E5-expressing cells form a highly abnormal epithelium. Many cells produce matrix metalloproteases (MMPs), which are characteristic of cells derived from the basal membrane. Based on a mutational analysis, the first hydrophobic domain of E5 was found to be required for viral invasion. Notably, E5-induced viral invasion might substantially contribute to tumor progression in persistently infected cervical epithelial cells, but this supposition remains to be confirmed [86]. The precise role of E5 in neoplastic transformation has not yet been fully elucidated, indicating a broad spectrum of research areas.

### 2.4. Effect of HPV Proteins on Signaling Pathways

The E5, E6, and E7 oncoproteins alter multiple signaling pathways in the initiation and maintenance of HPV-associated cancers. We distinguish a variety of mechanisms through which HPV may impinge cellular pathways for its own needs, such as p53, pRB, epidermal growth factor receptor (EGFR), PI3K/Akt/mTOR, JNK/ERK/AP-1, ERK, E-cadherin, Wnt/β-catenin, NF-kB, JAK/STAT, TGF-β/TNF-α, Hippo, MAPK HIF1/VHL/VEGF, EMT, and YY1 [87,88]. HPV can also interact with miRNAs that play regulatory roles in cell growth, apoptosis, cell migration, and metastasis. Below, we include an overview of the main pathways affected by HPV oncoproteins.

#### 2.4.1. p53

**Figure 4:**
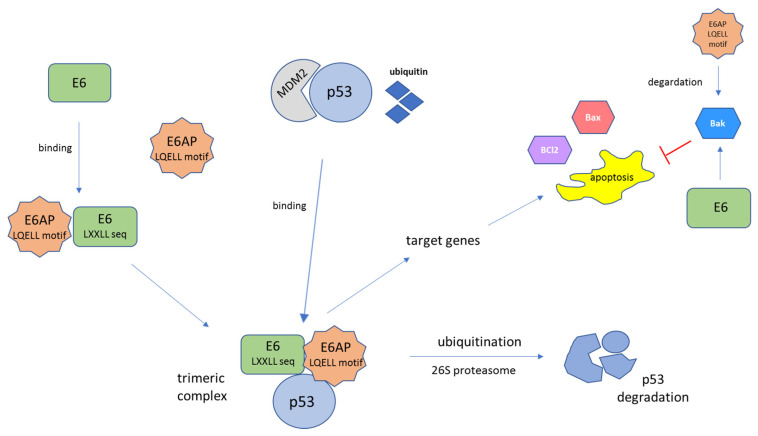
Schematic representation of E6-mediated tumor suppressor p53 protein degradation. The E6 protein, through a conserved binding motif containing the sequence LXXLL, binds to the LXXLL motif (LQELL) on the cellular E3 ubiquitin ligase E6AP. E6AP, E6, and p53 bind to each other and form a trimeric complex. This is followed by ubiquitin-dependent proteasomal degradation of the p53 protein. The polyubiquitinated p53 is then degraded by the 26S proteasome complex. The result of p53 degradation is the elimination of the trophic sentinel response to viral DNA synthesis and an increase in telomerase activity, leading to uncontrolled cell proliferation [94]. The function of E6 is the proteolytic inactivation of certain proapoptotic factors, such as p53, Bak, or Bax, through the ubiquitin–proteasome pathway. E6 can interact with Bak, Bax, and BCl2 directly, leading to the degradation of Bak in vivo. Moreover, E6 may block the Bak-mediated intrinsic mode of apoptosis through p53–E6AP interaction. Bak is also a target of the E6AP, while E6 stimulates the ubiquitin-mediated degradation of Bak through its interaction with Bak and E6AP [97]. MDM2—mouse double minute 2 homolog, a transcriptional target of p53.

The p53 protein is a well-characterized tumor suppressor protein often called the “guardian of the genome”. This protein is a key regulator of cell fate under stress conditions and acts as a transcription factor for the genes needed for apoptosis or cell cycle arrest [89]. The HPV E6 protein interacts with cellular proteins, thereby activating a number of oncogenic pathways that lead to blockage of senescence and apoptosis (Figure 4). The cellular mouse double minute 2 homolog (MDM2), also known as a transcriptional target of p53, was found to act as an E3 ubiquitin ligase, which transfers ubiquitin (Ub) to p53, thereby targeting it for proteasome-mediated degradation. The HR-HPV E6 oncoprotein interacts with target cellular proteins via a conserved binding motif containing the LXXLL sequence. In the host cell, a trimeric complex composed of E6, p53, and the cellular ubiquitination enzyme E6AP is formed. E6 binds to the LXXLL motif (LQELL) of the cellular E3 ubiquitin ligase E6AP and forms a heterotrimeric triplex of E6/E6AP/p53 [90,91]. Degradation of p53 occurs through ubiquitination with E6AP by the 26S proteasome [92,93]. When p53 is degraded, it does not induce either the growth arrest or the apoptosis of virus-infected cells. While both HR-HPV and LR-HPV E6 proteins can bind to the p53 C-terminus, only HR-HPV E6 proteins are capable of binding to the core region of p53, which is required for its degradation [94]. The perturbation of the p53 function by E6 causes destabilization of the host genome and uninterrupted cellular proliferation and is one of the critical factors in the neoplastic transformation of epithelial cells [94]. When the p53 protein is absent, cell division is uncontrolled, exhibiting checkpoint evasion. Some in vivo experiments have shown that the interaction of p53 with E6-AP is fundamental for the development of tumorigenicity in several types of tumors [95,96].

#### 2.4.2. pRB

**Figure 5:**
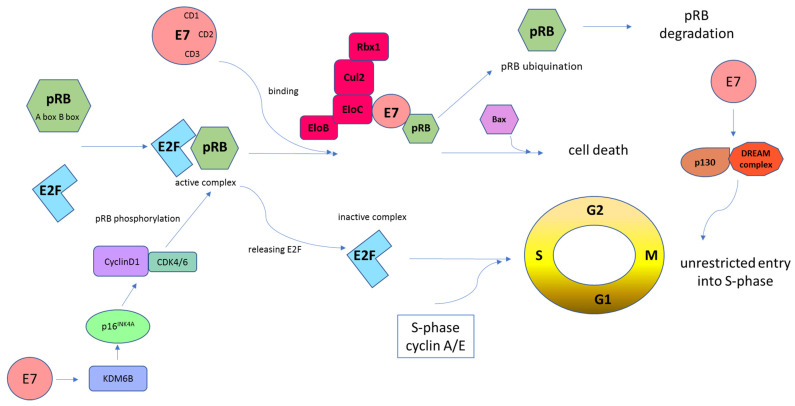
Schematic representation of E7-mediated retinoblastoma tumor suppressor protein (pRB) inhibition. Cells must pass the G1 restriction point, which is under the control of pRB, to progress from the G1 to the S cell cycle phase. E2F transcription factors are bound and repressed by pRB via the A and B boxes. Then, this complex binds the HR-HPV E7 protein. E7, through the CD2 and CD3 regions, binds to the B box of pRB via its LXCXE motif. Following these interactions, the pRB–E2F complex is disturbed, which leads to abnormal cell progression into the S-phase of the cell cycle. HPV E7-mediated pRB degradation can be mediated by the cullin 2 (Cul2) ubiquitin ligase complex. This interaction occurs via the E7 CR1 domain and the C-terminal sequences and drives cell cycle progression by degradation of pRB and upregulation of CDK2 and cyclins A/E. In addition, the cyclin D1/CDK4/6 complex phosphorylates pRB, which promotes E2F release. Subsequently, cyclin A/E facilitates pRB phosphorylation, allowing S-phase entry. This whole mechanism leads to unrestricted entry into the S-phase and unrestrained cell proliferation [55,76]. The E7 oncoprotein causes the transcriptional induction of KDM6B and, as a result, the p16INK4A expression. Induced expression of p16INK4A results in a G1 cell cycle arrest by inhibiting phosphorylation of pRB by CDK4/6 kinases [101]. Moreover, E7 targets p130 specifically in the DREAM complex to remove the barrier to entry into the S-phase [103,104].

The pRB protein is a tumor suppressor that is involved in the negative control of the cell cycle and in tumor progression [55]. This protein can bind the E2F transcription factor family and repress gene transcription required for transition from G1 to uncontrolled S-phase [55,76]. HR-HPV E7 oncoprotein inhibits pRB activity and disrupts its association with E2F (Figure 5). E2F activity is controlled through its association with pRB and two other pRB-related proteins, p107 and p130; they are responsible for the inhibition of cyclins A/Cdk2 and E/Cdk2 [98,99]. To prevent cells from entering the S-phase too early, the pRB protein remains bound to E2F. When encountering HPV-infected cells, E7 leads to pRB ubiquitination, which releases the E2F transcription factors. E2F transcribes cyclin E, cyclin A, and p16INK4A, an inhibitor of CDK4/6 that drives cells through premature S-phase entry [71,100]. The tumor suppressor p16INK4A protein is a significant target of HPV E7 during cell cycle regulation. The E7 oncoprotein upregulates the expression of p16INK4A through pRB disintegration and by epigenetic depression through KDM6B (H3K27-specific demethylase 6B) [100]. E7-mediated KDM6B induction accounts for the expression of p16INK4A. Induced expression of p16INK4A inhibits CDK4/6 activity [101]. The E7 oncoprotein also interacts with the DREAM complex, which is responsible for the repression of cell cycle-related genes [74,102,103]. The DREAM complex consists of E2F4, DP1 and p130/p107 in addition to RBBP4 and the LIN proteins that form the MuvB core. The p130 protein is an important target for E7 in promoting S-phase entry. It was found that E7 proteins from both HR-HPVs and LR-HPVs share an ability to target p130 for degradation [103,104]. The HPV16 E7 protein binds to the p130/DREAM complex through the LXCXE motif in the p130 pocket [74]. Then, the p130 protein is degraded by the ubiquitin–proteasome pathway. HPV16 E7 interferes with the p130/DREAM complex during the G0/G1 phase to promote the S-phase of the cell cycle. The B-myb/DREAM complex must be activated to express genes required in the S/G2/M phase [102]. HPV16 E7 is able to induce the proteasomal degradation of p130 and the related pocket proteins in keratinocytes [74,105]. The E7-mediated disruption of the DREAM complex seems essential for cell cycle progression [77,103]. 

#### 2.4.3. EGFR

The epidermal growth factor receptor (EGFR) is a 170 kDa transmembrane glycoprotein receptor that is encoded by the *Her-1* protooncogene located on chromosome 7p12. It is activated by the binding of some ligands, including the epithelial growth factor (EGF), giving rise to the formation of homodimers. EGFR functions through dimerization that activates a tyrosine kinase domain to regulate multiple functions such as cell growth, differentiation, gene expression, and development [106]. High EGFR expression is associated with tumor development and poor prognosis in cervical cancer.

**Figure 6:**
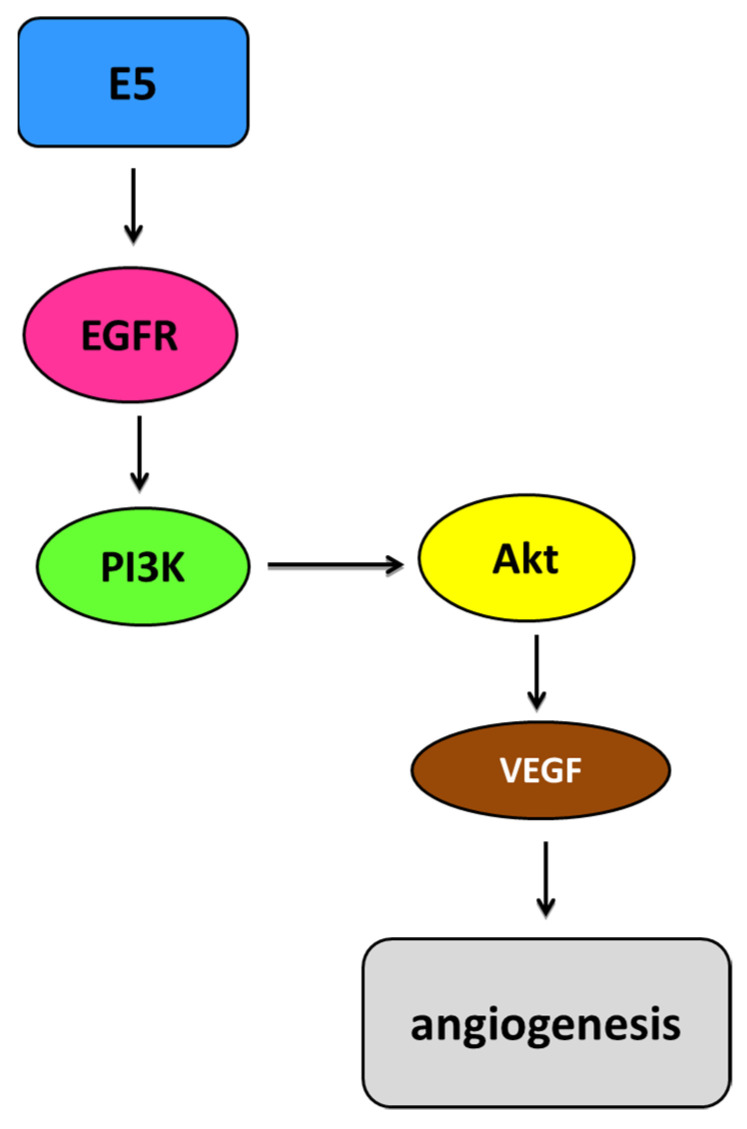
Simplified representation of the EGFR pathway mediated by E5. HPV E5 is involved in the activation of and increase in the epidermal growth factor receptor (EGFR) pathway depending on the ligand. Activated EGFR homodimers autophosphorylate, leading to increased activation of EGFR-related pathways such as the phosphoinositide 3-kinase (PI3K)/protein kinase B (Akt) pathway. Akt can affect upregulation of the vascular endothelial growth factor (VEGF), which consequently increases angiogenesis [113].

The HPV E5 oncoprotein is involved in the activation of and increase in the EGFR pathway. It interacts with the EGFR, the PDGF, and the colony-stimulating factor (CSF) and might promote angiogenesis in cancer through the EGFR/VEGFA pathway and the metastasis of HPV-containing malignancies. E5 creates complexes with the EGFR in cells overexpressing the receptor. This promotes prolonged activation of ERK1/2 and protein kinase B (Akt) in response to the EGF. The activation of EGFR-dependent pathways such as phosphoinositide 3-kinase (PI3K)/Akt increases the expression of the VEGF, leading to increased angiogenesis [107] (Figure 6). Disturbances in endocytosis have been observed in cells overexpressing the E5 protein, e.g., in the transport from early to late endosomes [108]. The E5 protein is associated with numerous morphological changes related to the reorganization of the actin cytoskeleton.

E5 can decrease the autophagy process by downregulating keratinocyte growth factor receptor/fibroblast growth factor receptor 2b (KGFR/FGFR2b) signaling activation and plays a role in the regulation of programmed cell death [109]. Through these effects, genetic mutations accumulate in cells with abnormal DNA, which promotes the malignancy process. E5 inhibits apoptosis by increasing the ubiquitination and proteasomal degradation of the proapoptotic protein Bax [110]. It has been shown that the E5 oncoprotein may play a key role in HPV-induced cancers, especially in the metastatic process, by upregulating the expression of *MET* transcripts and the hepatocyte growth factor receptor (HGFR) [111,112]. This upregulated expression leads to the extensive progression of lesions and lower patient survival.

#### 2.4.4. JAK/STAT 

**Figure 7:**
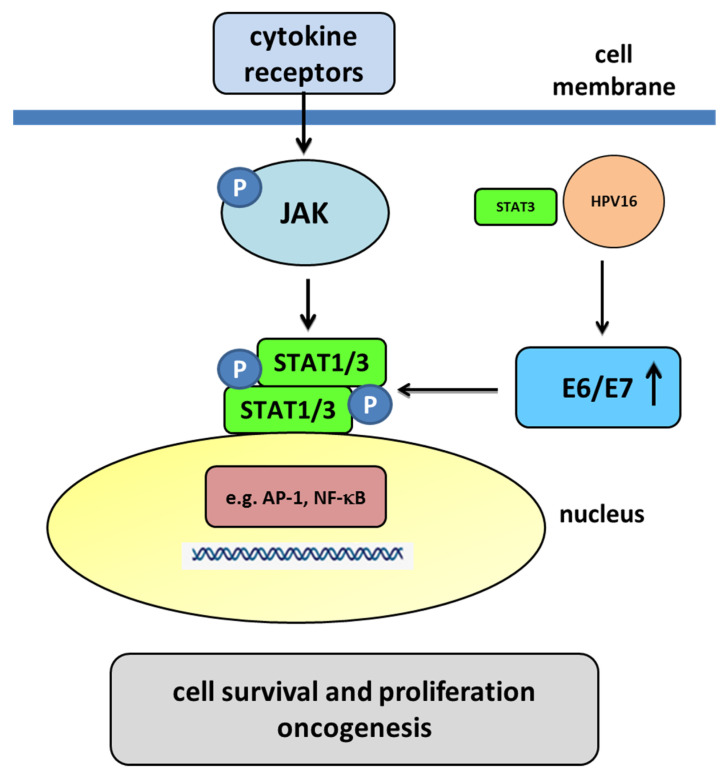
The JAK/STAT pathway mediated by the E6 and E7 oncoproteins. Membrane cytokine receptors have cytoplasmic tails in which inactive JAKs associate constitutively. The cytokine interaction with their receptors induces dimerization of these receptors. Interaction between the cytokine and its receptor results in the juxtaposition of JAKs, leading to their autophosphorylation. The activated JAKs then phosphorylate the receptor’s cytoplasmic tails on tyrosine residues, creating sites that allow the binding of other signaling molecules, such as STAT proteins. Cytoplasmic STATs bind to phosphorylated receptors, becoming substrates for the JAKs, which phosphorylate STATs on highly conserved tyrosine residues. After their phosphorylation, the STATs form homodimers or heterodimers that are capable of translocating to the nucleus and activating gene transcription. The E6/E7 oncoproteins decrease the translocation of STAT1 to the nucleus. A decrease in STAT1 is necessary for the amplification of the viral genome in the early stages of infection, meaning that STAT1 plays a protective role in the early phase of HPV infection. In the nucleus, transcription factors such as AP-1 and NF-κB, as well as STAT3 may play a regulatory role in HPV infection. HPV-infected cells produce large amounts of IL-6 for autocrine signaling and for increasing STAT3 activation. Some studies have suggested that STAT3 could bind to HPV16 upstream of the URR, driving the expression of E6/E7. Activated STAT3 results in an increase in the E6 and E7 oncoproteins. The oncoproteins promote a decrease in pRB and p53, which are the proteins that are responsible for the inhibition and arrest of the cell cycle and the promotion of apoptosis. HPV16 oncogenes downregulate the expression of IFN-responsive genes and upregulate proliferation-associated and NF-κB-responsive genes in cervical keratinocytes [121,130].

The Janus kinase (JAK)/signal transducer and activator of transcription (STAT) signaling pathway plays a key role in immune responses, cell proliferation, differentiation, and survival (Figure 7). The overexpression and overactivation of the components of the JAK/STAT pathway are associated with the development of different types of cancer, for example, in cervical cancer development [114]. The E5, E6, and E7 proteins may also be related to the JAK/STAT signaling pathway in cervical cancer. Firstly, we started by considering the role of STAT1 in cervical cancer, especially in cervical lesions. Some studies found STAT1 overexpression in cervical intraepithelial neoplasia (CIN) 1/2, a decrease in CIN3/cervical carcinoma in situ (CIS), and a significant increase in invasive cancers [115]. Significantly higher levels of STAT1 are observed in cervical cancer samples compared to other nontumor tissues [116]. Moreover, there are reports regarding the effect of HPV on STAT1. In HPV-infected human keratinocytes, both E6 and E7 oncoproteins independently suppress the expression of STAT1 [117]. The HPV16 E6 oncoprotein may reduce the amount of STAT1 and may also bind to the interferon (IFN)-stimulated response elements. Moreover, E6 and E7 proteins could decrease the translocation of STAT1 to the nucleus, and the decrease in STAT1 is necessary for the amplification of the viral genome in the early stages of infection, which is perhaps due to its ability to suppress IFN-inducible genes, thus evading the immune system [117]. Some studies show that the expression of STAT1 is essential for the induction of death in tumor cells, and its higher levels in cervical cancer samples compared to resistant cases suggest that STAT1 may contribute to improved radiosensitivity [118,119]. These findings exhibit that STAT1 may have a dual role in HPV infection and tumorigenesis, playing a protective role in the early phases of HPV infection, but functioning as a protooncogene in the invasive stages. However, STAT3 and STAT5 probably have the most critical roles in the development of cervical cancer. They are essential for proliferation and survival, in addition to being highly associated with tumor malignancy. In cervical cancer, the presence and activity of STAT3 is associated with the malignancy of cervical lesions [120]. HR-HPV-positive cells show a higher amount of active STAT3 (pY705) compared to HPV-negative cells [121]. The level of active STAT3 is associated with the number of copies of the HPV genome. Furthermore, HPV-positive cervical tumor cells produce high levels of interleukin 6 (IL-6) for autocrine signaling and to increase STAT3 activation [122]. STAT3 is an essential regulator in cell transformation, and different viruses have strategies to stimulate its signaling and activation [123]. The expression of the HPV genome mainly depends on host transcription factors, and some transcription factors such as AP-1, NF-κB, and STAT3 might play a regulatory role in HPV infection due to the presence of its cis-related elements in the upstream regulatory regions (URRs) and its association with the level of carcinogenesis [124]. STAT3 could bind to HPV16 upstream of the URR, driving the expression of E7 [125]. A positive correlation of active STAT3 with HPV16 E6 and E7 oncoproteins has been found [121]. Different studies show the inhibition of STAT3 with the STAT3-specific siRNA as a consequence that leads to the reduction of E6 and E7 [126]. The decrease in STAT3 in cervical tumor cells has a drastic effect and induces an increase in the expression of cell cycle control proteins such as p21, pRB, and p53, showing a decrease in cyclin D1 expression with an increase in the induction of apoptosis, which is produced by a decrease in proapoptotic proteins and an increase in the activation of effector caspases [121,126]. To sum up, the inhibition of STAT3 in tumor cells results in a decrease in the E6 and E7 oncoproteins. The lack of these oncoproteins promotes an increase in pRB and p53, which are the proteins that are responsible for the inhibition and arrest of the cell cycle and the promotion of apoptosis. The HPV E6 and E7 oncoproteins play an important role in the activation of STAT3 and STAT5. E6 induces the phosphorylation of JAK2-activating STAT3 and STAT5 and increases their amounts in infected cells. An increase in the activation of both proteins correlates with the intensity of a lesion, and their silencing affects the decrease in viral oncoproteins [126,127,128,129].

#### 2.4.5. PI3K/Akt/mTOR

The E6 and E7 oncoproteins are related to the PI3K/Akt/mTOR pathway, a major cancer survival pathway [131]. PI3K regulates Akt and Rac-1. Akt has downstream targets that control cell proliferation, cell growth, cell mobilization, angiogenesis, and cell survival [132]. This pathway has been associated with increased cancer initiation, progression, metastasis, and drug resistance [133]. E6 activates this pathway through multiple mechanisms. E6 inactivates the PTEN tumor suppressor protein via PDZ proteins, leading to increased pAkt and cell proliferation [113,134]. 

**Figure 8:**
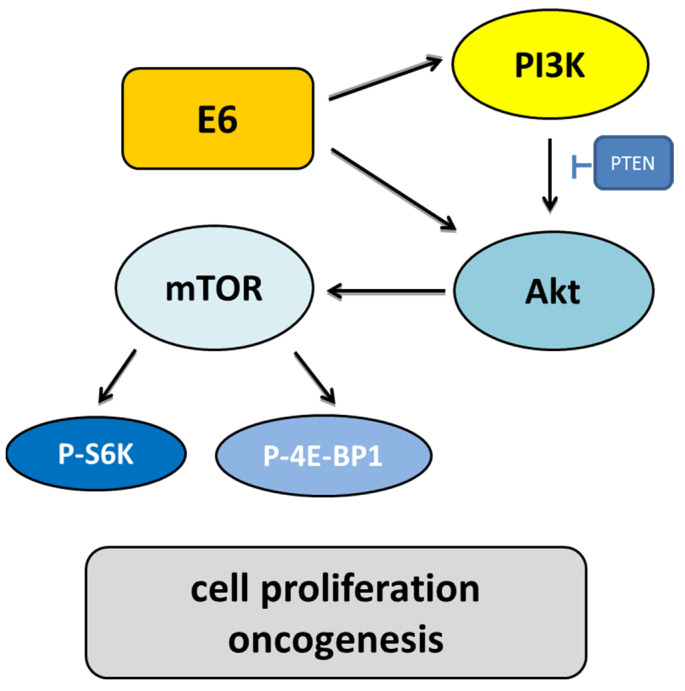
The role of the PI3K/Akt/mTOR signaling pathway induced by HPV infection. E6 activates PI3K through the receptor protein tyrosine kinase or direct interaction with PI3K. Throughout phosphorylation, PI3K activates Akt. Activated Akt influences cell growth through the mammalian targets of rapamycin (mTOR) and angiogenesis. Due to the upregulation of the ribosomal protein S6 kinase (S6K) and the blocking of the eukaryotic initiation factor 4E-binding protein (4E-BP), mTOR may increase cell proliferation. E6 also blocks tuberous sclerosis complex 1/2 (TSC1/2) to increase the mammalian target of rapamycin complex 1 (mTORC1) activity to increase cell growth and block the proapoptotic Bad and Bax proteins.

Another target of Akt is the mammalian target of rapamycin (mTOR) kinase (Figure 8). It has been demonstrated that mTOR complex 1 (mTORC1) is activated by E6, as indicated by increased levels of the ribosomal protein S6 kinase (S6K), which is regulated by mTOR [113]. It was initially thought mTORC1 could be activated by HPV16 E6-induced degradation of mTOR inhibitor tuberous sclerosis complex 2 (TSC2) in an E6AP-dependent manner [135,136]. Another study demonstrated that HPV16 E6 expression causes an increase in mTORC1 through enhanced phosphorylation of mTOR as well as activation of downstream targets—ribosomal protein S6 kinase (S6K) and eukaryotic initiation factor-binding protein 1 (4E-BP1) [136,137]. However, a decrease in TSC2 levels in HPV16 E6-expressing cells was not found [137]. Instead, HPV16 E6 expression causes Akt activation through upstream mTOR complex 2 (mTORC2) and putative 3-phosphoinositide-dependent kinase 1 (PDK1) [137]. E6 expression causes an increase in protein synthesis by enhancing translation initiation complex assembly at the 5′ mRNA cap and an increase in cap-dependent translation. HPV16 E6 activated mTORC1 through activating receptor protein tyrosine kinases, including the EGFR, the insulin receptor, and the insulin-like growth factor receptors [138]. This hyperactivity was confirmed to contribute towards PI3K/AKT/mTOR pathway activation in HPV16 E6-expressing foreskin keratinocytes.

Akt activation may produce a cascade of changes in later targets. Akt phosphorylates E6 to promote its ability to interact with the protein 14-3-3σ, which is a key step in carcinogenesis. HR-HPV E6 comprise C-terminal PBMs, which are subjected to phosphorylation events that are prone to modulating their interaction with PDZ domains and the 14-3-3 proteins [139]. Several studies show that E6 causes activation of the PI3K/Akt pathway and its other targets: nuclear factor-κB (NF-κB), mTOR, 14-3-3σ, and c-myc, but the effects of E6 on the other downstream targets of Akt have not been studied in detail [140,141,142,143,144,145]. 

#### 2.4.6. Wnt/β-Catenin

The Wnt signaling pathway is another HPV-related cancer pathway. Wnt ligands and the associated pathway regulate cellular proliferation and differentiation processes and play critical roles in normal tissue homeostasis [146], as well as in cancer development [147]. Wnt pathway activation results in the accumulation of β-catenin, which in turn increases the transcription of a broad range of genes to promote cell proliferation. When the Wnt pathway is inactivated, β-catenin forms a complex with other proteins, including glycogen synthase kinase-3β (GSK3β), casein kinases, adenomatous polyposis coli, and axin2, and is phosphorylated at serine and threonine residues. The phosphorylation of β-catenin induces its ubiquitination by the β-TcRP ubiquitin ligase, leading to degradation. However, when Wnt is in the activation status, intracellular protein is phosphorylated and interacts with Axin2, leading to the dysfunction of the degradation complex and the accumulation of β-catenin. The accumulated β-catenin is translocated into the nucleus and binds members of the T cell factor/lymphoid enhancer factor family of transcription factors to regulate target genes, including c-jun, c-myc [148], cyclin D1 [149], multidrug resistance 1 [150], matrilysin [151], axin2 [152], survivin, VEGF, COX-2, and matrix metalloproteinases [153]. In human HPV16-positive invasive cancer samples and in early dysplastic lesions, it is common to find accumulated nuclear β-catenin. Nuclear β-catenin accumulation can activate the Wnt pathway using HPV oncogenes [154,155]. Studies on cervical cancer show that the nuclear accumulation of β-catenin correlates with tumor progression in cervical cancer patients. The accumulated β-catenin also correlates with HPV infection in the cervical cell lines SiHa with bearing-integrated HPV16 and HeLa with bearing-integrated HPV18 [156]. Silencing the *E6* gene in HPV-positive cells reduced nuclear β-catenin substantially. These results suggest that E6 plays a critical role in the activation of the Wnt pathway. Studies have also shown that HPV16 E6 activates the Wnt/β-catenin pathway. The mechanism is independent of the ability of E6 to target p53 for degradation or bind to the PDZ-containing E6 targets but requires E6AP [157]. In vivo experiments showed that E6 expression led to the accumulation of β-catenin. The Wnt pathway could be a possible mediator for increased β-catenin [158]. PI3K/Akt is also known to cause the accumulation of β-catenin through inactivation of GSK3β [159]. 

#### 2.4.7. TLR Signal Transduction Pathways. Evading Immune Response

HPV has a prolonged replication period and must persist in the host epithelium without being detected for an extended period. Therefore, the virus develops complex mechanisms to escape host immunosurveillance and interfere with the host’s virus eradication mechanism [160,161]. HPV is detected by pattern recognition receptors (PRRs) that activate specific signaling cascades to induce the expression of target genes, including the activation of genes encoding type I IFNs and proinflammatory cytokines. It was reported that the HR-HPV type affects the PRR- and type I IFN-induced signaling pathways by downregulating the expression of IFN-stimulated genes (ISGs) [162]. Among PRRs, toll-like receptors (TLRs), especially TLR4 and TLR9, have been extensively studied in cervical cancer and have been positively correlated with HPV16 infection [163]. It was found that TLR9 recognizes HPV16 CpG-rich DNA, but its transcription is hindered by the E6 and E7 oncoproteins [164]. Suppressed TLR9 expression was observed in the cervical epithelium of women with HPV16-positive lesions compared to that of healthy women [164,165]. It was observed that TLR9 downregulation was associated with HPV16 E6 and E7 expression in keratinocytes and in cervical cancer-derived cell lines [166]. Moreover, TLR9 downregulation affects IFN response, which negatively regulates HPV16 infection [167]. In contrast, the HPV18 oncoproteins are not able to reduce TLR9 levels [167]. The activity of the oncoproteins affects the ability to recognize pathogens, thereby enabling the virus to escape from immune surveillance. E6 participates in direct immune system modulation by binding to interferon regulatory factor 3 (IRF-3) and by downregulating its transcriptional activity. The HR-HPV E6 and E7 proteins deregulate the activity of the NF-κB pathways [168,169,170,171]. This downregulation decreases the expression of type I IFNs and proinflammatory cytokines that create an immune response against viral antigens [172,173]. It is suggested that NF-κB plays a protective role during the early phases of HPV infection and persistence while promoting tumor progression in advanced lesions [174]. HR-HPV E6 also attenuates retinoic acid-inducible gene I (RIG-I)-mediated signaling by promoting the ubiquitination and degradation of TRIM25, thus dampening type I IFN production [175].

The E6 oncoprotein may inhibit phosphorylation of tyrosine kinase 2 (TYK2) through the signal transducer and activator of transcription (STAT/TYK2) pathway and therefore prevents the association between IFN type α (IFNα) and its receptor [176]. Upon E6-dependent hypermethylation, the keratinocyte-specific IFN type κ (IFNκ) is downregulated, and then STAT1 expression is inhibited. The same process affects proapoptotic protein superfamily member 10 (TNFSF10) and, more specifically, TLR3 expression and XIAP associated factor 1 (XAF1) [177,178]. The E7 oncoprotein disrupts IFN signaling by binding to IRF1 and IRF9 [179,180,181]. The HPV16 E5 protein may dysregulate the expression of IFN by suppressing STAT, leading to the suppression of downstream ISGs in keratinocytes [182]. Moreover, the E7 oncoprotein may block a key component of the innate immune system, affecting cytoplasmic DNA and the cyclic GMP–AMP synthase–stimulator of interferon genes (cGAS–STING) activity [183]. Both the E6 and E7 oncoproteins induce tumor-associated inflammation by upregulating the expression of proinflammatory cytokines, including IL-6 and IL-8 [184,185,186]. As a consequence, the inflammatory process in the infected cell is initiated, which leads to the upregulation of metalloproteinases, proangiogenic factors, and chemokines that can support tumor progression [187]. The E6 and E7 oncoproteins are critical for triggering the angiogenic switch in HPV-induced cancers by downregulating the angiogenic inhibitors mammary serine protease inhibitor (Maspin) and thrombospondin-1. In addition, E6 and E7 may upregulate the expression of VEGF [186]. HPV also evades the host immune response by perturbing the expression of HLA class I and II molecules. HPV16 oncoproteins have been reported to downregulate MHC-I expression [41,188,189,190]. E5 interacts with MHC-I by inhibiting the transportation of molecules in the Golgi apparatus to the cell surface. This mechanism leads to the complex having a decreased ability to present viral antigens to CD8^+^ T cells [41,190]. The downregulation of cell surface molecules allows the virus to establish persistent infection.

## 3. Mechanisms of HPV-Mediated Oncogenesis in HPV-Related Gynecological Cancers

HPV infection is one of the main causative factors in female genital tract cancers. Epidemiological studies show that HPV is detected in almost all cervical cancers and in between 40–85% of all vaginal and vulvar carcinomas. Considering that HPV is the most common sexually transmitted disease worldwide, the role of this oncovirus in the development of HPV-driven cancers is exaggerated. Prophylactic HPV vaccines against HR-HPVs are expected to offer protection against precursor lesions and genital carcinomas.

### 3.1. Cervical Cancer

Cervical cancer comprises the following histologic subtypes: squamous carcinoma (SCC), adenocarcinoma, and adenosquamous carcinoma. The majority (75%) of cervical cancers are of the SCC type. The old term for cervical dysplasia, “cervical intraepithelial neoplasia” (CIN), was changed to “squamous intraepithelial lesion” (SIL) following the 2001 revision of the Bethesda classification [191]. CIN1 is classified as a low-grade squamous intraepithelial lesion (LSIL), while CIN2 and CIN3 are classified as high-grade SILs (HSILs) and show positive results for HR-HPV types having the potential to progress into invasive cancer [192]. Over 70% of HSILs and cervical SCCs are associated either with HPV16 or HPV18 infections. In women younger than 40 years old, HPV infection was found in 89% of adenocarcinomas, while in women over 60 years old, it was found in 43% of adenocarcinomas [193]. It was suggested that HPV16 infection is preferentially associated with SCCs and adenocarcinoma, while HPV18 is mainly a risk factor for the development of adenocarcinoma [194]. 

HPV infection may induce changes from a normal to dysplastic cellular architecture in the transformation zone. Most neoplastic lesions develop from cells in the transition zone, which is defined as the border between the cervical surface squamous epithelium and the glandular epithelium of the cervical canal. Small injuries expose the basal layer cells to HPV-infected cells from the cervical epithelium, enabling its penetration, which is a receptor-mediated process. The molecules that are involved in this process may be the heparin sulfate proteoglycans and integrins (α6, β1, and β4) that are present in the basal cell epithelium [62,63]. When basal cells are infected with HPV, they divide, stay in the basal layer, and retain their dividing ability, acting as a store for viral replication. When the HPV genome is delivered to the nucleus, the expression of the early HPV genes *E1* and *E2* is launched. These genes activate viral replication by expropriating cellular DNA replication factors [195,196]. To ensure that cervical cells continue to constantly grow and divide, early HPV genes *E5*, *E6*, and *E7* are expressed, stimulating cells to propagate and grow. After cellular differentiation in the suprabasal layer, *E4*, *L1*, and *L2* expression is activated. Subsequently, a capsid around the virus’ genomic material is formed by the L1 and L2 proteins, and the mature viral particles are released from the epithelial cells. The virions are sloughed off with the dead squamous cells of the epithelium.

HR-HPVs often integrate their genome into the human genome in cervical SSC tissue samples [197,198,199,200,201]. Genome integration can be an early event in the progression of LSILs to HSILs. HPV genome integration into the host genome is observed in 50–80% of HPV16-positive and almost all HPV18-positive cases of cervical SCCs, although in approximately 15% of cases, the virus remains in the episomal form [197,198,199,200,201]. It was demonstrated that integration can take place within the MYC locus [202]. Hence, *c-Myc* expression is often altered in HPV-infected cervical cancer cells. Integration frequently leads to the disruption of the *E2* gene site and the expression of the *E6* and *E7* oncogenes. However, HPV integration per se does not necessarily lead to increased oncogene expression or a cell growth advantage [203]. The E6 and E7 oncoproteins disrupt cell cycle checkpoint control by degrading cell cycle regulators and inhibiting CDKs inhibitors (p21, p27). As described earlier, the E6 and E7 proteins contribute to achieving uncontrolled proliferation through deregulation of growth suppressors. In HPV-infected cervical cancers, oncoproteins manage to express the human telomerase reverse transcriptase (hTERT). Both E6 and E7 can activate the hTERT promoter via a c-Myc-dependent mechanism, thus contributing to the immortality of cancer cells [144,204,205]. E6 induces the hTERT promoter via interactions with E6AP and with the c-Myc and NFX1 proteins [206]. Deregulation of c-Myc expression leads to disruption of E2F, Cdks, and cyclins. Myc is further found to reverse the Cdk-inhibiting activity of p21 and p27 [207]. Neither addition of hTERT nor induction of telomerase activity by E6 results in immortalization. Inactivation of the Rb/p16 pathway by E7 or downregulation of p16 expression, in combination with telomerase activity, can immortalize epithelial cells efficiently [208]. Biomarker p16 shows intense and continuous staining in HSILs and suggests infection with an HR-HPV type [209]. However, a small subset of HPV-associated cervical carcinomas does not overexpress p16. These p16-negative cases are detected in older women and are associated with a worse prognosis [210,211].

### 3.2. Vaginal Cancer

Histologically, about 90% of vaginal cancers are squamous cell carcinomas (VaSCC) [212]. Vaginal cancer could be preceded by vaginal intraepithelial neoplasia (VaIN), a precursor lesion caused by HPV exposure of unknown prevalence and progression rate. Diagnosis of primary vaginal cancer is rare because most of these lesions (approximately 80–90%) are metastatic from another primary site. 

Several studies examined the prevalence of HPV infection in VaIN and vaginal cancer [213,214,215,216]. HPV infection is an important risk factor for vaginal carcinoma, especially in countries with high HIV prevalence. Cofactors include mainly immunosuppression and cigarette smoking. HPV DNA has been detected in 55–81% of invasive vaginal cancers depending on the detection method used [217,218,219,220]. A meta-analysis revealed that overall HPV prevalence in VaSCCs was 69.9% and 93.6% in VaIN cases [217]. In another study, a pooled prevalence of HPV was 67% in VaSCCs and 85% in VaINs [220]. HPV16 was the most frequently detected type in both lesions, followed by HPV18, HPV33, and HPV31 [215,217]. Persistent HPV16 infection is associated with long-term development of HSILs and carcinomas of the vagina [221,222]. 

When HR-HPV DNA is integrated into host cell DNA, its carcinogenic effect on vaginal epithelial cells is exerted through the viral oncoproteins E6 and E7, which are actively transcribed. The E7 oncoprotein binds to and inactivates pRB, affecting cell cycle control, overturning the control and repair system in the cell, and leading to an overexpression of the tumor suppressor protein p16, a cyclin-dependent kinase-4 inhibitor. A meta-analysis of the prevalence of p16 revealed that the vast majority of HPV-positive vaginal cancers show p16 overexpression, suggesting active involvement of the virus in the malignant transformation process [222]. 

### 3.3. Vulvar Cancer

HPV is also known to cause some vulvar squamous cell cancers (VSCCs). The precursor lesion for VSCC is high-grade vulvar intraepithelial neoplasia (VIN). Approximately 25–42% of VSCCs are induced by HR-HPVs [215,223,224]. In contrast to non-HPV-associated cancer that is considered a rapidly progressing lesion, HPV-associated VIN develops slowly and is associated with a favorable prognosis [225]. A meta-analysis comprising 5015 cases of vulvar cancer and 2764 cases of VIN revealed that the prevalence of HPV in vulvar cancer was 39.7% and 76.3% in VIN lesions [226]. Basaloid and warty variants of VSCC are more common in younger women, are often associated with HPV DNA detection [227], and get similar risk factors as in cervical cancer. HPV prevalence in invasive basaloid and warty tumors is more frequent (69.4%) than in invasive VSCC types observed in elderly women (13.2%) [215]. HPV-associated vulvar neoplasia is mostly associated with the HPV16 type; however, some other HPV types, including 18, 33, 45, and 52, may play a role in vulvar carcinogenesis [215,224,226,228]. Infection with HR-HPVs and viral DNA integration into the host cell genome seems to be related to the progression of VIN and its key steps are similar to those described in cervical cancer [229]. Recently, it has been recommended to document the HPV status of vulvar carcinomas (HPV-associated or HPV-independent) [227]. This is assessed by p16 block-type immunoreactivity and/or positive molecular testing for HPV. 

### 3.4. Uterine (Endometrial) Cancer

Endometrial cancer is one of the most common gynecological malignancies affecting more than 300,000 women worldwide. Its incidence is much higher in more developed countries. The involvement of HPV infection in the pathogenesis of endometrial carcinoma is controversial. A meta-analysis examining the prevalence of HPV DNA in tumor tissue from endometrial cancer revealed that the pooled prevalence of HPV DNA in endometrial cancer was 10.0% [230,231,232,233]. However, the HPV prevalence varied considerably from 0% to 54.5% [230]. Among the tissues of 25 endometrial adenocarcinomas, 24% were HPV16-positive and 20% were HPV18-positive [231]. It appears that the presence of HPV in the endometrium seems to have a limited role in the etiology of endometrial cancer despite the close anatomical proximity to the cervix. However, the mechanisms of HPV-mediated carcinogenesis in endometrial cancer have not been investigated yet.

### 3.5. Ovarian Cancer

Ovarian cancer (OC) is the eighth most common cancer in women around the world. OC is a heterogeneous disease with different histological types. Serous tumors account for approximately 70% of all epithelial ovarian cancer (EOC) tumors and are responsible for the majority of deaths from ovarian cancer. More than 90% of serous carcinomas are very aggressive and defined as high-grade serous ovarian carcinomas (HGSOCs). The remaining 10% are low-grade serous ovarian carcinomas (LGSOCs) that generally have a better prognosis. Asymptomatic early stages of the disease and failure to identify precursor lesions delay tumor detection, which makes diagnosis difficult until the OC is in advanced stages [234,235,236].

Three hypotheses have been formulated to explain how both the fallopian tube and the ovary might contribute to tumorigenesis of EOC. The first hypothesis suggests that the border between the fallopian tube and the ovary is an area of epithelial transition that is rich in stem cells and therefore vulnerable to malignant transformation [237]. The second theory suggests that malignant tubal epithelial cells (from serous tubal intraepithelial carcinoma (STIC)) can be implanted on the exposed surface of the ovary, resulting in the formation of secondary tumors [238]. In addition, HGSOC was thought to arise from the ovarian surface epithelial (OSE) cells or cortical inclusion cysts [239,240]. Many HGSOCs of the pelvis are thought to originate in the distal portion of the fallopian tube. A transcriptome analysis revealed that most HGSOCs more closely resemble normal fallopian tube epithelium (FTE) than the OSE [241,242]. Nevertheless, up to 12% of HGSOCs show greater transcriptional similarity to the OSE [241]. The evidence has suggested that both FTE and OSE cells are likely precursors of HGSOC [240,241,242,243]. FTE-derived and OSE-derived tumors differ in the transcriptome, latency, and metastatic behavior [243]. Ovarian carcinoma has also been associated with inherited risk mutations in the *BRCA1*, *BRCA2*, *RAD51C*, *RAD51D*, and *TP53* genes [244]. The risk factors for ovarian cancer are ovulation, chronic infection (pelvic inflammatory disease (PID)), and endometriosis [237,245]. The question of the potential influence of HPV on the development of ovarian cancer has not been answered univocally yet. Some studies confirmed the presence of HPV in malignant ovarian cancer [5,6,246,247,248,249,250,251,252,253,254] and fallopian tube specimens [5,6]. In contrast, other experiments did not confirm the presence of HPV in EOC [255,256,257]. HR-HPV 16 and 18 were the predominant types detected in patients with OC [5,6,246,247,248,249,250,251,252,254,258,259,260]. Other viral types, including HPV33, HPV6, and HPV45, as well as mixed HPV infections were also reported [6,246,247,253,258]. Two meta-analyses, published in 2013 and 2021, demonstrated that the pooled HPV prevalence in OC tissue worldwide was approximately 16%, but wide geographical variation (from 0% to 81%) was found [261,262]. The highest pool prevalence of HPV in OC cases was reported in Asia (45.6% and 30.9%) and Eastern Europe (18.5% and 29.3%), the lowest—in North America (0%) [261,262], respectively. Our study showed the presence of HPV DNA, mostly the HPV16 type, in the majority of cancerous ovarian tissues [6]. It should be noted that viral infection was low-grade and only detected by highly sensitive techniques. HR-HPV viral integration or the expression of viral oncogenes by detection of *E6* and/or *E7* mRNA was also detected [251,263]. HPV DNA detection and overexpression of the p16 protein were found in 32.3% of EOC cases [263]. However, the prognostic significance of p16 is highly variable in OC studies [264]. 

It is hypothesized that the inactivation of p53 and pRB by the HR-HPV E6 and E7 oncoproteins can lead to the development of ovarian cancer. However, the mechanisms that can induce the development of disease in patients remain unknown. The neoplastic processes in EOC may be similar to those found in other types of cancer, e.g., cervical cancer, and research is still ongoing to confirm or refute this possibility and create a completely new model of EOC development. Taking into consideration the fatal course of ovarian cancer and the lack of very effective treatment in advanced and recurrent cases, it is important to understand the mechanisms of its formation. Based on the proven role of HPV in many cancers, it seems reasonable to check a possible role of HPV in ovarian carcinogenesis. This speculation is supported by observations that inflammation plays an important role in triggering and maintaining EOC progression. Moreover, it was observed that tubal closure reduces the risk of EOC (probably by cutting off the route of ascending infection) [265]. 

## 4. Conclusions

HPV can cause multiple types of gynecological cancers, although HPV is the greatest risk factor for cervical cancer. A subset of cancers, including vulvar and vaginal cancers, have been attributed to HR-HPV infection. The role of HPV in ovarian and endometrial cancers is still undefined. The most widely accepted hypothesis points to occasional HPV infection in combination with other risk factors, including chronic inflammation and genetic predisposition. HPV infection plays an important role in the risk of precancerous lesions and therefore in the dysplastic and malignant transformation of lesions. The main factor influencing carcinogenesis is the presence of HPV oncoproteins. These viral proteins have a remarkable capacity to impair multiple key regulatory pathways and induce all of the known characteristic features associated with cancers. The E6 oncoprotein can inactivate p53 and PDZ while stimulating the PI3K/Akt and Wnt pathways. E7 can inhibit pRB and stimulate the PI3K/Akt pathway. E5 can augment their function and contribute to tumor progression. Altered signaling pathways in turn promote cell proliferation, decrease in cell apoptosis, increase in angiogenesis and cell migration. The combined expression of the HPV E6 and E7 oncoproteins leads to a complementary and synergistic effect that induces cell immortalization and transformation. Understanding the mechanism of HPV involvement in cancer development and the role of viral oncoproteins is essential for the effective prevention of the neoplastic process and the development of screening tests and therapies. 

## Figures and Tables

**Table 1:** The properties and functions of HPV proteins.

Protein Name	Molecular Weight	Number of Amino Acids	Function	References
E1	73 kDa	649	− DNA helicase − initiation of viral DNA replication − forms a replication complex with E2 − binds to the p62 protein subunit of the human homolog of transcription factor TFHII and the p80 protein − interaction with DNA polymerase alpha	[31,32,33]
E2	42 kDa	365	− initiation of viral DNA replication − transcription factor − interaction with the E1 protein, bromodomain-containing protein 4 (Brd4), DNA topoisomerase II-binding protein 1 (TopBP1) − control of early region viral gene expression	[34,35]
E4	10.5 kDa	92	− involves cell cycle arrest − disruption of keratin filaments − expressed as an E1^E4 transcript	[36,37,38]
E5	9.4 kDa	83	− transmembrane protein − localized in the Golgi apparatus − regulates growth signaling pathways via activation of the epidermal growth factor receptor (EGFR), downstream regulation of the Ras–Raf–MAP kinase pathway or the PI3K–Akt pathway − proliferation of the altered cell − inhibition of apoptosis due to the tumor necrosis factor-related apoptosis-inducing ligand (TRAIL) and the Fas ligand (FasL) − prevents formation of the death-inducing signaling complex (DISC) induced by TRAIL − interaction with MHC/HLA class I − stimulation of interferon β1 (IFNβ1) and interferon regulatory factor 1 (IRF-1) − interaction with other oncoproteins − interaction with vacuolar ATPase, platelet-derived growth factor (PDGF), zinc transporter ZnT1, protocadherin 1 (PCDH1)	[39,40,41,42,43,44,45]
E6	18–19.2 kDa	150	− oncoprotein − p53 protein degradation − upregulation of the expression of hTERT telomerase − interaction with caspase 8 − cell cycle deregulation − collaborative action with the E7 protein leads to malignant transformation − interaction with the Bak protein − interaction with the Bax protein − interaction with E6AP ubiquitin ligase that is essential for E6 stability − interaction with HECT domain-containing ubiquitin ligase EDD − interaction with proteins containing PDZ domains	[19,46,47,48,49,50,51,52,53,54]
E7	11 kDa	98	− oncoprotein − pRB inactivation − interaction with centromere protein C (CENP-C) − functional inactivation permitting cell progression to the S-phase of cell cycle − uncontrolled cell division − collaborative action with the E6 protein leads to malignant transformation − interaction with cellular non-receptor protein tyrosine phosphatase PTPN14 − interaction with the pRB-related members of the pocket protein family like p107 and p130 involved in cell cycle regulation − association with the 600 kDa retinoblastoma protein-associated factor (p600) − association with members of the cullin 2 (Cul2) ubiquitin ligase complex	[55,56,57,58,59,60,61]
L1	55 kDa	531	− major capsid protein of a virus-like particle (VLP) − binding of the basal membrane of keratinocytes substantively to heparin sulfate proteoglycans (HSPG) − attends in the mechanism of viral entry by binding to the α6 integrin	[62,63]
L2	74 kDa	462	− minor protein of the capsid − promotes the transportation of the virion into the nucleus of the host cell by interacting with host dynein following endosomal entry − interaction with the Kapα2β1, Kapβ2, and Kapβ3 receptors − mediates the egress of the viral genome from endosomes	[9,30,64,65]
